# Macrophage Migration Inhibitory Factor (MIF) Promotes Increased Proportions of the Highly Permissive Th17-like Cell Profile during HIV Infection

**DOI:** 10.3390/v14102218

**Published:** 2022-10-09

**Authors:** César Trifone, Lucía Baquero, Alejandro Czernikier, Paula Benencio, Lin Leng, Natalia Laufer, María Florencia Quiroga, Richard Bucala, Yanina Ghiglione, Gabriela Turk

**Affiliations:** 1 Facultad de Medicina, Universidad de Buenos Aires, Buenos Aires C1121ABG, Argentina; 2 Instituto de Investigaciones Biomédicas en Retrovirus y SIDA (INBIRS), CONICET—Universidad de Buenos Aires, Buenos Aires C1121ABG, Argentina; 3 Departamento de Química Biológica, Facultad de Ciencias Exactas y Naturales, Universidad de Buenos Aires, Buenos Aires C1121ABG, Argentina; 4 Department of Medicine, Yale University School of Medicine, New Haven, CT 06510, USA; 5 Departamento de Microbiología, Parasitología e Inmunología, Facultad de Medicina, Universidad de Buenos Aires, Buenos Aires C1121ABG, Argentina

**Keywords:** HIV, MIF, MDM/CD4TL co-culture, immune pathogenesis

## Abstract

In this study, we evaluate the role of the MIF/CD74 axis in the functionality of CD4^+^ T lymphocytes (CD4TL) during HIV infection. MDMs from healthy donors were infected with a R5-tropic or Transmitted/Founder (T/F) HIV strain. At day 11 post-MDM infection, allogeneic co-cultures with uninfected CD4TLs plus MIF stimulus were performed. Cytokine production was evaluated by ELISA. MIF plasma levels of people with HIV (PWH) were evaluated by ELISA. The phenotype and infection rate of CD4TLs from PWH were analyzed after MIF stimulus. Intracellular cytokines and transcription factors were evaluated by flow cytometry. Data were analyzed by parametric or non-parametric methods. The MIF stimulation of HIV-infected MDMs induced an increased expression of IL-6, IL-1β and IL-8. In CD4TL/MDM co-cultures, the MIF treatment increased IL-17A/RORγt-expressing CD4TLs. Higher concentrations of IL-17A in supernatants were also observed. These results were recapitulated using transmitted/founder (T/F) HIV-1 strains. The MIF treatment appeared to affect memory CD4TLs more than naïve CD4TLs. MIF blocking showed a negative impact on IL17A^+^CD4TL proportions. Higher MIF concentrations in PWH-derived plasma were correlated with higher IL-17A^+^CD4TL percentages. Finally, MIF stimulation in PWH-derived PBMCs led to an increase in Th17-like population. MIF may contribute to viral pathogenesis by generating a microenvironment enriched in activating mediators and Th17-like CD4TLs, which are known to be highly susceptible to HIV-1 infection and relevant to viral persistence. These observations establish a basis for considering MIF as a possible therapeutic target.

## 1. Introduction

Infection by the human immunodeficiency virus (HIV) remains as a major public health concern worldwide. The hallmark of untreated infection includes persistently high viral load (VL), decline in CD4 T-lymphocyte (CD4TL) counts and systemic immune activation, ultimately leading to the development of acquired immunodeficiency syndrome (AIDS) [1]. Combined antiretroviral therapy (cART) reverts these effects by interfering with viral replication; it diminishes VL to undetectable levels, reducing not only morbidity and mortality of people with HIV (PWH) but also transmission risks, with the subsequent impact on the dynamic of the global epidemic [2]. However, cART has several limitations, such as life-long dependence, the need of daily doses, the development of viral resistance and toxicity. Additionally, even in PWH under suppressive treatment, a high rate of morbi-mortalities not related to AIDS is observed. Complications associated with chronic inflammation, immune dysfunction, intestinal mucosa disruption, hepatic dysfunction and monocytes/macrophages activation are developed and point to the fact that even effective cART does not restore a health status similar to uninfected individuals [3,4].

More importantly, it is widely known that plasma VL levels inevitably rebound in most PWH who discontinue cART. This evidences the presence of long-lived viral reservoirs that are resistant to cART and not recognized by the immune system, hampering the cure of the infection [5]. Thus, understanding the mechanisms underlying HIV persistence and irreversible immune damage is extremely important to fight the infection and its consequences. Moreover, the development of remission or cure strategies will depend on a deeper knowledge of these mechanisms [6].

CD4TLs are the main cellular target for HIV infection. This population is very heterogenous and each subset is endowed with a distinct phenotype and functions [7]. In particular, the expression density of the HIV receptor molecule CD4 and co-receptors (CCR5 and CXCR4), varies between CD4TL subsets. Relevant to this study, the Th17 subset highly expresses both co-receptors, making this population one of the most susceptible to HIV infection [8], which is also enhanced because of the particular gene expression pattern of this cell type [9], making it an important source of new virions during productive infection. What is more, viral cell-associated DNA has been readily detected in Th17 and Th1/Th17 cells in samples obtained from PWH under suppressive cART [9]. These, and other properties reviewed by Fromentin et al. [5], support the ability of Th17 cells to serve as a long-lived viral reservoir for HIV.

Monocytes and macrophages are also important actors during HIV infection, because they are targets of the infection but also because they trigger by-stander mechanisms upon activation. Viral DNA can be detected in multiple tissues such as liver, lung, central nervous system (CNS) and intestine locations where macrophages constitute a stable population and act as a key factor in maintaining tissue homeostasis and architecture. Infected macrophages are an important source of new virions due to their great capacity of resisting HIV cytopathic effects. In addition, macrophages are capable of sustaining viral production under effective cART and even in the absence of CD4TL. All these aspects have been recently reviewed [10,11].

Macrophage migration inhibitory factor (MIF) is a multifaceted cytokine produced by a broad variety of cell types in different tissues. It is involved in innate immune response regulation, anti-stress and anti-bacterial response, promoting pro-inflammatory functions on immune cells. On the other hand, it has been reported that MIF is upregulated and plays a role in infectious diseases, inflammatory diseases such as sepsis, asthma, arteriosclerosis, lupus, acute respiratory distress and numerous cancer types [12,13]. Particularly, MIF plasma levels are augmented in PWH, during acute infection and remain high despite effective cART [14,15]. Additionally, it was demonstrated that the addition of MIF promotes viral replication on HIV-infected PBMCs [14,15,16]. MIF mediates its function mainly after binding to an heteromeric receptor formed by CD74 (invariant chain or li) and other proteins, such as CD44 or chemokine receptors CXCR2 and CXCR4 [17,18,19], promoting inflammatory, anti-apoptotic and proliferative processes. In HIV-infected cells, it has been observed an upregulation of CD74 expression due to Nef viral protein activity as an inhibitor of the AP2-dependent endocytic pathway [20].

In this context, we have been working under the hypothesis that the positive Nef-mediated modulation of CD74 in HIV-infected cells plus MIF overexpression play a relevant role in HIV-mediated immune dysfunction and immunopathogenesis. In this line, previous reports of our group demonstrated that CD74 upregulation may have a physiological function in vivo as a correlation between CD74 surface expression on HIV-infected monocyte-derived macrophages (MDMs) obtained from PWH and the expression of activation markers on CD4TLs was observed [21]. Additionally, we have described that CD74/MIF interaction triggers the production of soluble inflammatory factors by primary HIV-infected MDMs generating a pro-inflammatory environment and enhancing non-activated CD4TL permissiveness [16]. As an extension of these works, in this paper, we aim to evaluate how MIF interaction on HIV-infected MDMs modulates CD4TL polarization, favoring skewing towards a Th17-like phenotype.

## 2. Materials and Methods

### 2.1. Primary Human Monocyte-Derived Macrophages (MDMs) and CD4^+^ T-Lymphocyte (CD4TL) Purification and Culture

Buffy coats from healthy donors (HD) were used to obtain peripheral blood mononuclear cells (PBMCs) by Ficoll-Hypaque (GE Healthcare Life Sciences, Marlborough, MA, USA) density gradient centrifugation. HDs were eligible voluntary blood donors > 18 years old who completed a survey on blood donation, which particularly excludes people who have been exposed to HIV and were screened for serological markers before being accepted as donors. Monocytes were then separated from PBMCs by Percoll (GE Healthcare Life Sciences, USA) gradient technique. Isolated monocytes (purity > 80% measured by flow cytometry) were further purified by adherence to plastic flat-bottom plates in RPMI 1640 medium (HyClone, Logan, UT, USA, GE Healthcare Life Sciences, USA). Non-adherent cells were removed after 1 h plating by means of extensive washes with PBS 1X. Adherent cells were allowed to differentiate into MDMs in RPMI 1640 medium supplemented with 10% fetal bovine serum (FBS, Gibco, Thermo Fischer Corporation, Waltham, MA, USA), 2 mM L-glutamine (Sigma-Aldrich, St. Louis, MO, USA), 100 U/mL penicillin (Sigma-Aldrich, St. Louis, MO, USA), 100 µg/mL streptomycin (Sigma-Aldrich, St. Louis, MO, USA) and 10 mM HEPES (Sigma-Aldrich) (from now on complete RPMI medium) plus 20 ng/mL recombinant granulocyte-macrophage colony-stimulating factor (GM-CSF, Miltenyi, Bergisch Gladbach, Germany) during 4 days. After differentiation, MDM purity was analyzed by flow cytometry and only cultures with >90% purity were used in subsequent assays. CD4TLs were isolated from buffy coats by negative selection using the RosetteSep kit (Stem Cell, Vancouver, BC, Canada).

### 2.2. Virus Production and Infections

Viral stocks of different viral variants were used across the study. First, a R5-tropic HIV-1 viral stock was produced by infecting primary MDMs from healthy donors with the HIV-1 BAL strain. Additionally, viral stocks of pseudotyped transmitted/founder (T/F) viruses were produced by co-transfecting 293T cells with plasmids encoding infectious molecular clones (IMCs) selected from the full panel of T/F IMCs available at the NIH AIDS Reagent program (Division of AIDS, NIAID, NIH: Cat #11856 (R5-tropic T/F virus, from now on Virus 3, V3), cat #11742 (X4-tropic T/F virus, from now on Virus 4, V4), cat #11744 (dual X4- and R5-tropic T/F virus, from now on Virus 6, V6), cat #11746 (R5-tropic T/F virus obtained after an event of heterosexual transmission, from now on Virus 8, V8), and Cat #11747 (R5-tropic T/F virus obtained after an event of male-to-male transmission, from now on Virus 9, V9) from Dr. John Kappes [22,23] together with a plasmid encoding the vesicular stomatitis virus (VSV) protein G, using the X-treme GENE 9 DNA transfection reagent.

Culture supernatants were harvested 48 h post-transfection (for T/F viruses) or 14 days post-infection (for the BAL R5-tropic stock). In all cases, culture supernatants were clarified by centrifugation at 600 g for 15 min at 4 °C, fractioned and stored at −80 °C until use. Viral titter was estimated by p24 antigen quantitation by ELISA (Sino Biological Inc., Beijing, China).

Monocyte-derived macrophages were infected with the BAL R5-tropic virus using a ratio of 2 ng p24/106 cells, or with the T/F strains with a ratio of 150 ng p24/106 cells. Infection was left to proceed for 11 days, when different stimuli were applied and cocultures with CD4TLs were performed. When noted, infection percentage was evaluated by p24 intracellular staining analysis by flow cytometry.

At day 11, MDMs were washed twice with PBS 1X (Sigma-Aldrich, St. Louis, MO, USA) and rhMIF was added to a final concentration of 25 ng/mL or 100 ng/mL. Non-stimulated cultures were used as negative control. Cells were incubated at 37 °C and at 8, 72 or 120 h the supernatants were harvested, whereas the cells were recovered at 72 and 120 h only.

### 2.3. MDM/CD4TL Co-cultures

Allogenic purified CD4TLs (>95% purity by flow cytometry) were co-cultured with HIV-infected MDMs in complete RPMI, in a 1:1 ratio and maintained for 5 days. Simultaneously to the co-culture initiation, different stimuli were added: recombinant human MIF (rhMIF, 25 ng/mL or 100 ng/mL), neutralizing MIF antibody (αMIF antibody, 100 ng/mL), MIF098 MIF antagonist (100 nM), the corresponding isotype control (100 ng/mL) or a combination of IL-6 and IL-1β neutralizing antibodies (20 ng/mL αIL-6, 180 µg/mL αIL-1β); the non-stimulated control was a co-culture maintained on complete RPMI alone.

CD4TLs, MDMs and culture supernatants were harvested at day 3 and 5 post-co-culture. CD4TLs were recovered from the plate with culture supernatant and separated by centrifugation at 1500 rpm for 5 min. When noted, MDMs were washed twice with PBS 1X, harvested after treatment with Triple Express trypsin (Gibco, EE.UU.) and surface expression of CD80 (αCD80 PE, clone L307.4, BD Biosciences, Franklin Lakes, NJ, USA), CD86 (αCD86, clone 2331 (FUN-1), BD Biosciences) and HLA-DR (αHLA-DR, clone L243, BioLegend, San Diego, CA, USA) was evaluated by flow cytometry.

### 2.4. Recombinant Cytokines and Antibodies

Recombinant human MIF (rhMIF) was prepared as described elsewhere [24] (endotoxin content  < 0.1 EU/mL). MIF antagonist MIF098 [3-(3-hydroxybenzyl)-5-methylbenzooxazol-2-one] was dissolved in DMSO at a concentration of 149 µM [25]. The neutralizing anti-MIF monoclonal antibody (clone NIHlllD.9) was obtained from ascites after purification using protein A/G spin column and resuspended at 5.15 mg/mL [26,27]. The cytokine neutralizing antibodies anti-IL-6 and anti-IL-1β (BioLegend, San Diego, CA, USA) were obtained.

### 2.5. Cytokine Quantitation

The levels of the following cytokines were evaluated in MDM supernatants using commercially available kits: IL-8, IL-6, IL-1β, TNFα, IL-10, IL-17A (ELISA MAX Deluxe kits, BioLegend), sICAM (DouSet ELISA, R&D Systems), IL-4, IL-2 and IFNγ (Th1/Th2 CBA kit, BD). MIF plasma levels were evaluated using an in-house ELISA constructed with an anti-human MIF antibody pair and an MIF standard obtained from BioLegend.

### 2.6. CD4TL Phenotype, Viability and Infection Percentage

In order to evaluate CD4TL activation, the cell surface expression of CD38 and HLA-DR was analyzed by flow cytometry after co-culture with infected MDMs. CD4TLs were harvested and stained for CD3 (αCD3-PerCP, clone SK7, BioLegend), CD4 (αCD4-APC, clone RPA-T4, BD Biosciences), CD38 (αCD38-BV650, clone HB-7, BioLegend) and HLA-DR (αHLA-DR-FITC, clone L243, BioLegend). Percentages of cells expressing these markers as well as their mean fluorescence intensity (MFI) were recorded. Initial gating was performed on lymphocytes followed by gating on CD3^+^CD4^+^ events (Figure S1A). Isotype-matched non-specific antibodies were used in each sample to set the corresponding negative populations accurately.

In addition, CD4TL viability, phenotype and infection percentages were evaluated at day 5 post-co-culture by flow cytometry. First, cells were incubated in the presence of Brefeldin A (2 µM, BioLegend, San Diego, CA, USA) and Monensin (2 µM, BioLegend, San Diego, CA, USA). After 6 h, CD4TLs were harvested and stained for surface molecules CD3 and CD4. Intracellular staining of IL-17A (αIL-17A-PE, clone BL168, BioLegend), IFNγ (αIFNγ-Bv421, clone 4S.B3, BD Biosciences) and p24 (αp24, clone KC57, Beckman-Coulter, Brea, CA, USA) was performed using the Cytofix/Cytoperm kit (BD Pharmingen), according to the manufacturer’s instructions. In certain experiments, the expressions of RORγt (α RORγt-AlexaFluor647, clone Q31-378, BD Biosciences) and Tbet (αTbet-PerCP-Cy5.5, clone 4B10 BioLegend) were evaluated after cellular permeabilization with FoxP3 Fix/Perm buffer set (BioLegend, San Diego, CA, USA). Data acquisition was performed in a BD FACSCanto flow cytometer using the BD FACSDiva software and analyzed subsequently with FlowJo v10 software (Data Analysis Software, LLC). To determine cell viability, single cells were gated in an FSC-H versus an FSC-A plot. Then, living lymphocytes were gated an FSC-A versus an SSC-A plot (%viability). Subsequently, infected cells were identified in an CD3 versus p24-FITC plot (Figure S1B). Alternatively, infection was estimated by p24 antigen quantification in culture supernatants by ELISA (Sino Biological Inc., Beijing, China).

### 2.7. Quantitative Real-Time PCR for Cell-Associated (CA) HIV DNA

CA HIV-integrated DNA was quantitated by real-time PCR. CD4^+^ T cells were isolated from frozen PBMCs using an immunomagnetic selection kit (Miltenyi Biotec, Bergisch Gladbach, Germany; purity > 95%). Cell pellets were resuspended in a lysis buffer (10 mM Tris-HCl, pH 8.0; 1 mM EDTA; 10 mg/mL proteinase K; Invitrogen) and digested for one hour at 55 °C in a heating shaker, follow by proteinase K inactivation at 95 °C for 5 min [28]. Cell lysates were quantified and stored at −80 °C until use. HIV-integrated DNA was measured as described in [29,30] from cell lysates. Assays were run in triplicates and HIV DNA copy numbers were standardized to cellular equivalents using as housekeeping a CD3 real-time PCR. Quantitative real-time PCR assays were run for 40 cycles.

### 2.8. Naïve and Memory CD4TL Cell Sorting

When noted, sorted naïve and memory CD4TLs were used to perform co-cultures with HIV-infected MDMs instead of bulk CD4TLs. Starting from CD4TL purified by negative selection as described previously, naïve (CD3^+^CD4^+^CD45RA^+^CCR7^high^) and memory (CD3^+^CD4^+^CD45RO^+^CCR7^−^) CD4TLs were obtained by cell sorting using a BD FACS Fusion cell sorter (BD Biosciences, USA). The whole panel included staining of the following membrane markers CD45RO, CD45RA, CCR7, CD3, CD4 and CD8 in the pre- and post-sorting analysis. After co-culture with infected MDMs, proportions of IL-17A-expressing cells were evaluated as described above.

### 2.9. Human Samples from People with HIV

Plasma and PBMCs from 24 individuals with recent HIV-1 infection were obtained. These subjects were enrolled as part of an ongoing acute/early primary HIV infection cohort study from Argentina [30,31,32,33,34,35,36,37]. This study was reviewed and approved by two institutional review boards: Comité de Ética Humana, Facultad de Medicina, Universidad de Buenos Aires and Comité de Bioética, Fundación Huésped (Buenos Aires, Argentina). Samples from HDs were also obtained. Both HIV-infected participants and HDs provided written informed consents accepting to participate in this study in accordance with the Declaration of Helsinki.

Frozen PBMCs were thawed at 37 °C in a water bath and washed twice with 10 mL of PBS 1X plus 2% FBS, 1 mM of HEPES and 20 U/mL of DNAse. After that, cells were incubated at 37 °C in a humidified atmosphere with 5% CO_2_ during 6 h. Rested PBMCs were cultured in U-bottom 96-well plates at a density of 0.5 × 106 cells per well with complete RPMI medium plus 20 U/mL IL-2. PBMCs were also incubated with 50 or 100 ng/mL of rhMIF. Negative controls were cells without any stimulus, and for the positive and isotype control, cells were incubated with 20 ng/mL of αCD3/αCD28 antibodies (BD Biosciences). After 24 h, cells were harvested and proportion of IFNγ-like and IL-17A-producing cells were measured ex vivo as described in [38] and analyzed by flow cytometry. Supernatants were also harvested for cytokine quantification by ELISA.

### 2.10. Data Analysis

Experiments were performed independently at least three times and analyzed using parametric tests, unless otherwise stated (see exact number of independent experiments and tests in each figure legend). Data normality was assessed using the Shapiro–Wilk test. All tests were considered significant when *p* < 0.05 (GraphPad Prism 7 Software).

## 3. Results

### 3.1. Cytokine Expression by MIF-Treated HIV-Infected MDMs

**Figure 1:**
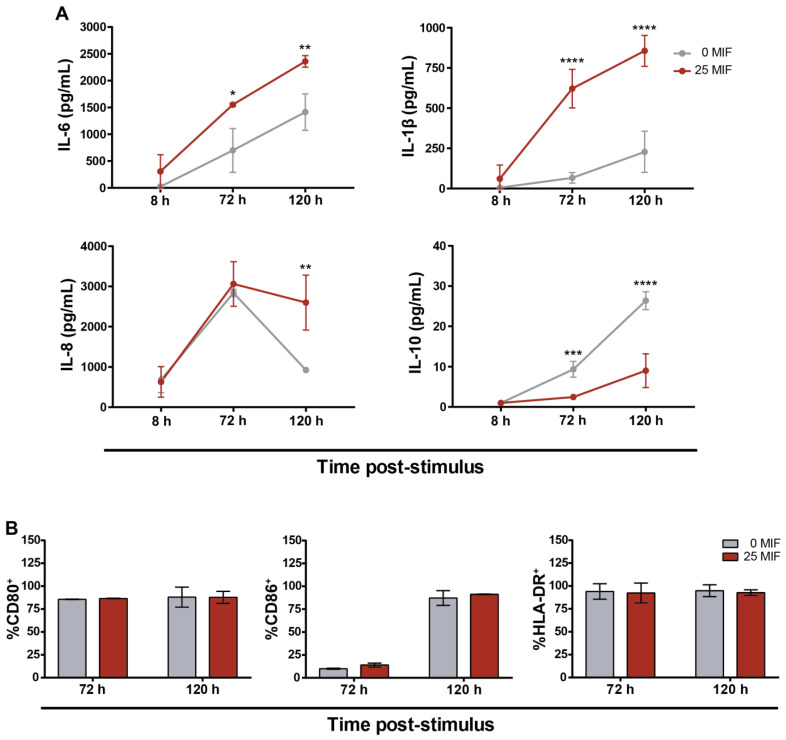
Activation status of HIV-infected MDMs after MIF stimulus. (**A**) Concentration of IL-6, IL-1β IL-10 and IL-8 in supernatants from HIV-infected MDMs treated (dark red lines) or not (grey lines) with MIF. (**B**) CD80, CD86 and HLA-DR surface expression on HIV-infected MDMs treated (dark red bars) or not (grey bars) with MIF after 72 and 120 h of stimulus. Data show the mean ± SD of three independent experiments each one performed in triplicate. Data analysis was performed by one-way ANOVA and Sidak’s post-test. * *p* < 0.05, ** *p* < 0.01, *** *p* < 0.001, **** *p* < 0.0001.

We have previously shown that MIF treatment on HIV-infected MDMs triggers the production of several proinflammatory cytokines in the short term (8 h) [16]. This is in line with MIF treatment producing the same effect on a wide variety of cells [13,39]. The evaluation of the cytokines released to extracellular media and membrane marker expression on MDM surface is indicative of its profile [40]. Thus, we started by evaluating MDM phenotype and production of proinflammatory markers after longer periods (8, 72 and 120 h) following MIF stimulus (25 ng/mL). Higher levels of IL-6, IL-1β and IL-8 and lower levels of IL-10 were detected in MIF stimulated condition, compared to untreated controls (Figure 1A). TNFα expression was not altered by MIF treatment and neither IL-4 nor IFNγ were detected in these supernatants. No differences in the surface expression of CD80, CD86 and HLA-DR were observed between MIF-treated and control conditions after 72 or 120 h post-stimulus (Figure 1B), and no differences were either observed when analyzing their MFI. Thus, the concentration of pro-inflammatory cytokines IL-6, IL-1β and IL-8 remained elevated 120 h post-MIF stimulus, while no difference was observed in the expression of MDM activation markers.

### 3.2. CD4TL Differentiation and Activation Profile after Contact with MIF-Treated HIV-Infected MDMs

During antigen recognition, the immune environment, defined as the concentration and proportions of different cytokines, is key in the profiling of the subsequent immune response. Thus, we wondered how the particular environment generated by MIF- treated HIV-infected MDMs would alter the differentiation of CD4TLs after activation. We chose an allogenic coculture model because it could mimic an inductive site. Infected MDMs would act as the antigen presenting cell, providing foreign antigens (MHC II mismatch) and co-stimulatory signals. The cytokine context and differentiation signal would be provided by MIF stimulus on infected MDMs. We specifically decided to study the Th17 profile based on previous observations where we identified that MIF–CD74 interaction on HIV-infected MDMs generated an environment enriched in IL-6 and IL-1β [16], leading us to hypothesize a possible Th17 polarization bias under those conditions. Proportions of Th1-like cells were also monitored as counterpart.

**Figure 2:**
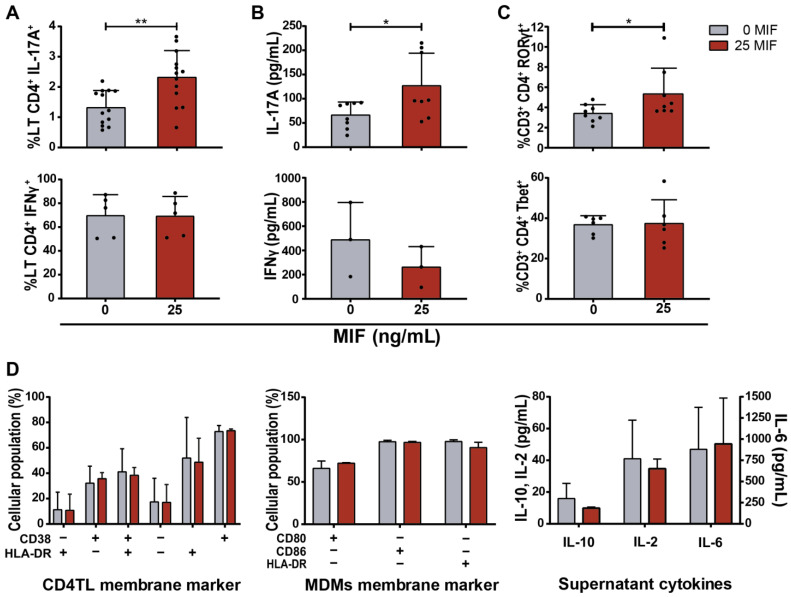
CD4TL differentiation profile after five days of co-culture with HIV-infected MDMs treated with MIF. (**A**) Proportions of IL-17A-producing CD4TL (upper panel) and IFN-γ-producing CD4TL (lower panel); (**B**) Concentration of soluble IL-17A (upper panel) and IFN-γ (lower panel); (**C**) Proportions of RORγT-expressing CD4TLs (upper panel) and T-bet-expressing CD4TLs (lower panel) in MIF-treated and untreated co-cultures. (**D**) Expression of activation markers CD38 and HLA-DR on CD4TLs, CD80, CD86 and HLA-DR on MDMs and concentrations of IL-2, IL-6 and IL-10 in culture supernatants. Dark red bars: MIF-treated cultures. Grey bars: untreated cultures. Each point represents an independent experiment comprising triplicates. Bars indicate mean ± SD of all independent experiments. Data analysis was performed by Student’s *t*-test. * *p* < 0.05, ** *p* < 0.01.

Five days after co-culture, higher percentages of CD3^+^CD4^+^IL-17A^+^ (Th17-like) events were observed in MIF-stimulated cultures compared to the untreated control (41% increase in average with respect to the unstimulated control) (Figure 2A upper panel, Figure S1C). However, no differences were found in the percentages of CD3^+^CD4^+^IFNγ^+^ (Th1-like) events (Figure 2A lower panel, Figure S1C). What is more, elevated concentrations of soluble IL-17A were found in MIF-treated cultures compared to the untreated cultures (Figure 2B upper panel). On the contrary, soluble IFNγ concentration showed a non-statistically significant decrease in culture supernatants when stimulated with MIF (Figure 2B lower panel). In order to reinforce the concept that MIF would be favoring the development of a Th17-like profile, the expressions of the transcription factors RORγt and Tbet (characteristic from the Th17 and Th1 profiles, respectively) [41] were analyzed on CD4TLs from eight and six donors, different from the previous ones. A significant increase in the proportion of CD3^+^CD4^+^RORγt^+^ events was found in MIF-treated cultures compared to the untreated control (Figure 2C upper panel, Figure S1D), while no changes were found in CD3^+^CD4^+^Tbet^+^ events (Figure 2C lower panel, Figure S1D). Neither CD4TLs nor MDMs showed differences in the expression of membrane activation markers between MIF-treated and untreated conditions (CD38 and HLA-DR, and CD80, CD86 and HLA-DR for CD4TLs and MDMs, respectively) (Figure 2D). This led us to think that the observed effect on CD4TL is specific of differentiation, while the activation level is not altered and that this effect cannot be attributed to differences in MDM activation between conditions. Finally, no differences were observed in the concentrations of soluble IL-10, IL-2 and IL-6 (Figure 2D).

### 3.3. CD4TL Trans-Infection by MIF-Treated HIV-Infected MDMs

**Figure 3:**
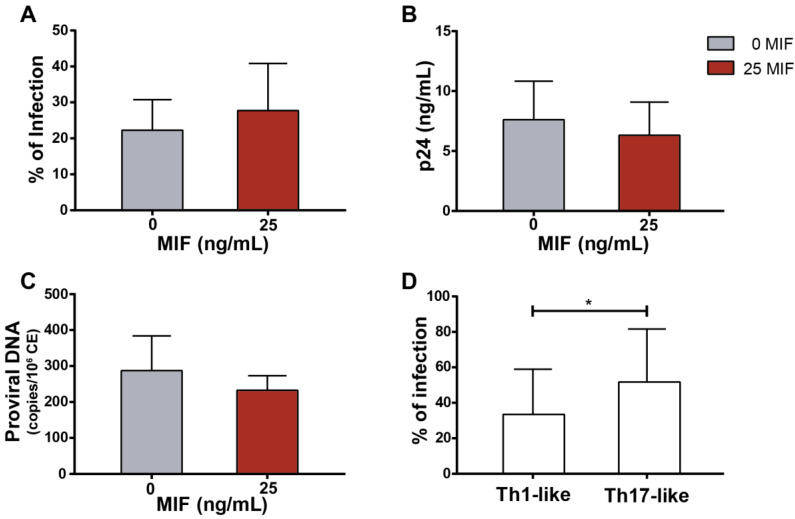
HIV infection on CD4TL and viral production on infected co-cultures. (**A**) Proportion of p24^+^ events within CD4TL bulk population. (**B**) p24 viral protein concentration in culture supernatants. (**C**) HIV DNA pro-viral DNA copies per million of cells within CD4TL bulk population. (**D**) Proportion of p24^+^ events within Th17-like and Th1-like CD4 T-cell populations. Data represent the mean ± SD of three independents experiments each one performed in triplicate. Data analysis was performed with Student’s *t*-test (* *p* < 0.05).

Since MDMs are infected and co-cultured with non-infected but permissive CD4TLs, CD4TL infection mediated by cell-to-cell transmission is a factor to be accounted. To evaluate the efficiency of this process in the presence of MIF, infection percentages were evaluated in CD4TLs after five days of co-culture by p24 antigen intracellular staining and flow cytometry analysis (Figure 3A), by evaluating the concentration of p24 antigen in culture supernatants (Figure 3B), and by quantitation of proviral DNA by real-time PCR (Figure 3C). No differences were found between the MIF-treated cultures and the untreated cultures, suggesting that MIF stimulation had no effect on cell-to-cell trans-infection process. As it is broadly described in bibliography, Th17 profile shows a higher susceptibility and permissiveness to HIV-1 infection compared to other CD4TL profiles. However, differences in the proportions of Th17-like cells shown in Figure 2 did not translate into a measurable difference in the efficacy of total cell-to-cell viral infection. However, as it is shown in Figure 3D, Th17-like cells in the cell culture presented an overall higher infection percentage compared to our co-culture model. Additionally, no differences in the infection percentage of Th17-like cells were observed when comparing the MIF stimulated condition to the untreated control. Thus, the results obtained to date might indicate that MIF would promote an increase in the number of Th17-like cells in this co-culture model, without increasing their susceptibility to HIV infection.

### 3.4. Soluble Factors Released after MIF Treatment in HIV-Infected MDMs Implicated in CD4TL Polarization

**Figure 4:**
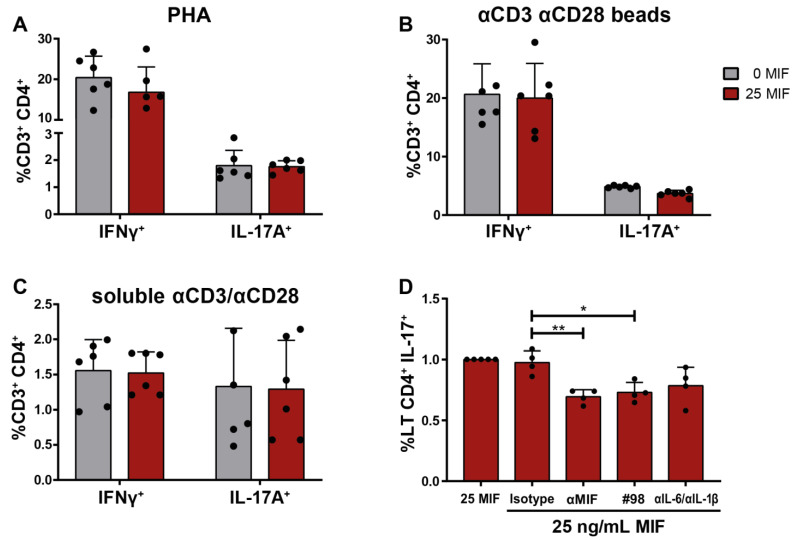
Study of the mechanism involved in Th17-like profile promotion. IL-17A and IFNγ-expressing CD4TLs after conditioning with HIV-infected MIF-stimulated MDM-derived supernatant in parallel with (**A**) PHA stimulation, (**B**) CD3/CD28-engaging beads stimulation and (**C**) CD3/CD28-engaging antibodies stimulation. (**D**) Proportion of IL-17A-producing CD4TLs after MIF blocking in the context of an MIF-treated cell co-culture. From left to right: cell co-culture control with 25 ng/mL of MIF, immunoglobulin isotype control, MIF-neutralizing monoclonal antibody, MIF chemical antagonist (#98), IL-6 and IL-1β neutralizing monoclonal antibodies. Each point represents an independent experiment comprising triplicates. Bars indicate the mean ± SD of all independent experiments each one performed in triplicate. Data analysis was performed by a one-way ANOVA and Tukey’s post-test (excluding the positive control). * *p* < 0.05, ** *p* < 0.01.

Conditions within the inductive sites are crucial for the polarization of the immune response, including CD4TL subsets. One special condition is the cytokine context, which determines the phenotype acquired by lymphocytes. As shown in Figure 1, MIF stimulus on HIV-infected MDMs led to the preferential expression of certain soluble factors, such as IL-6 and IL-1β, and the inhibition of others, such as the anti-inflammatory cytokine IL-10. To test if these three factors could be implicated in CD4TL profiling, non-activated CD4TLs were incubated with supernatants derived from MIF-treated HIV-infected MDMs. Activating signals, previously provided by the contact to MDMs, were replaced by external stimuli as phytohemagglutinin (PHA) (Figure 4A), beads coated with αCD3/αCD28-binding antibodies (Figure 4B) or soluble αCD3/αCD28-binding antibodies (Figure 4C). None of these conditions could replicate CD4TL polarization to Th17 profile in the presence of MIF stimulus. This points to a third or fourth factor that cannot be simulated properly in this new experimental model, which could be related to the co-stimulatory signals, TCR-mediated recognition and/or the viral transmission process that takes place after cellular contact.

To shed some light on the original hypothesis that the interaction of MIF with its receptor CD74 on HIV-infected MDMs leads to the release of cytokines that skew CD4TL profiling, a new co-culture was performed. To test this idea, αMIF-neutralizing antibody or MIF antagonist (both tools interfere with the receptor–ligand binding process as described in [26]) were added at the moment of MIF stimulus. Figure 4D shows that these tools significantly inhibited the activation profiling of CD4TL into a Th17-like phenotype. This result confirms that MIF is involved in the results obtained and also provides support to the notion that MIF interaction with CD74 might be specifically involved. It was also interesting to test if the released cytokines, such as IL-6 and IL-1β, were involved in the CD4TL profiling. Adding neutralizing antibodies that recognize IL-6 and IL-1β was presented as a possible way of inhibiting this pathway. Although a decrease in CD3^+^CD4^+^IL-17A^+^ events was observed after IL-6 and IL-1β neutralization, the effect was not statistically significant (Figure 4D). It is tempting to conclude that IL-6 and IL-1β might have a role in CD4LT profiling together with other factors, such as TGFβ, which was not quantified in this paper.

### 3.5. MDMs Infected with T/F HIV Strains Also Promote the Generation of a Th17-like Profile after MIF Treatment

**Figure 5:**
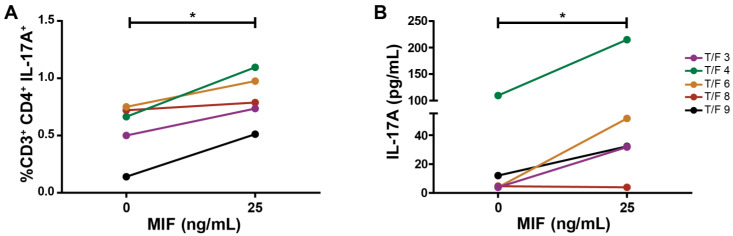
Transmitted/founder (T/F) HIV-strain MDM infection of co-culture resembles Th17-like profiling. (**A**) Proportion of IL-17A-expressing CD4TLs on MIF-treated co-cultures with T/F HIV-strain-infected MDMs. (**B**) Soluble IL-17A concentration in culture supernatants on MIF-treated co-cultures with T/F HIV-strain-infected MDMs. Data show the mean of two independents experiments with five different T/F HIV strains each one performed in triplicate. Data analysis was performed with a Student’s *t*-test (* *p* < 0.05).

Throughout all this research, we used only one laboratory HIV-1 strain, which could bias the results. Therefore, the experiments were replicated to include MDM infection with five different transmitted/founder HIV-1 strains. Figure 5A shows how the stimulus with MIF led to an increase in IL-17A-expressing CD4TL when MDM infection was performed with these clinically relevant HIV-1 strains. Additionally, the soluble IL-17A concentration was increased in culture supernatants of stimulated cultures, compared to control (Figure 5B). In light of this new evidence, it can be stated that the MIF-induced profiling of the CD4TL activation process occurs independently of the HIV-1 strain.

### 3.6. Profiling of Naïve and Memory CD4TLs by the Environment of HIV-Infected MIF-Treated MDMs

To date, we observed that MIF-treated HIV-infected MDMs provide an environment that favors bulk CD4TL differentiation into Th17-like cells. The translation of these results to an in vivo context would face some difficulties, regarding the limitation of MDMs as antigen-presenting cells and its physiological location. Although MDMs are professional antigen-presenting cells, they are reported as deficient in activating naïve CD4TLs because of the low expression of co-stimulatory molecules. Moreover, MDMs are not located in lymphoid tissues where naïve CD4TL activation takes place. In face of these two drawbacks, it is reasonable to think that naïve CD4TLs are not the main population affected by this profiling process. Infected MDMs could play a role in this process as a permanent population of peripheral tissues, where only effector CD4TLs arrive because of the inflammatory environment and chemotactic signals. In this context, identifying whether naïve or memory CD4TLs are more susceptible to the profiling process described in this work is highly relevant.

**Figure 6:**
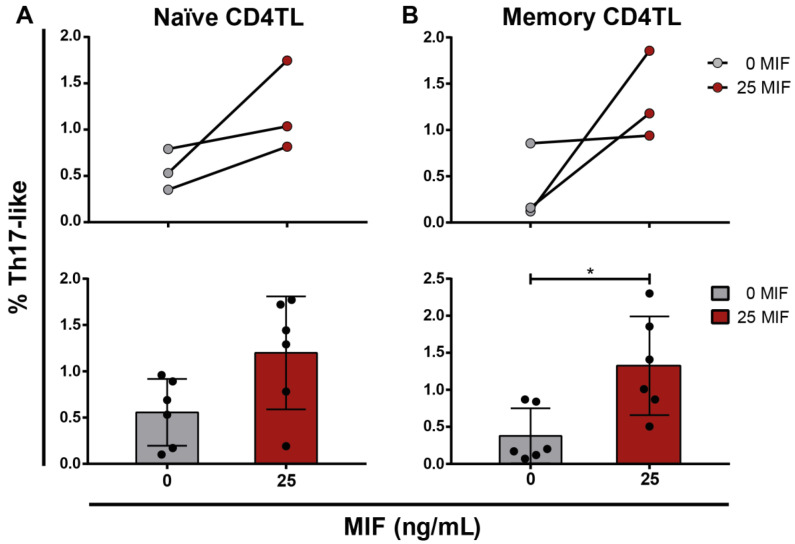
Study of the response of naïve and memory (CD4TL populations to MIF stimulus. Proportion of IL-17A expressing CD4TL in cell co-cultures performed with BAL R5-infected MDMs and: (**A**) sorted naïve CD4TLs (CD45RA^+^CCR7high) and (**B**) sorted memory CD4TLs (CD45RO^+^CCR7^−^). Individual experiments mean values (upper panel), all replicates (below panel). Data represent the mean ± SD of three independent experiments each one performed in triplicate. Data analysis was performed with a Student’s *t*-test (* *p* < 0.05).

Naïve (CD45RA^+^CCR7^high^) and memory (CD45RO^+^CCR7^−^) CD4TLs were sorted from non-activated bulk CD4TLs. Both populations were plated over HIV-infected MDMs and co-cultured for 5 days, with or without MIF. Then, cells were harvested and IL-17A expression was evaluated by flow cytometry. In the MIF-treated condition, higher proportions of cells with a Th17-like phenotype were found in both naïve (Figure 6A) and memory CD4TLs (Figure 6B). However, this increment was significantly different, compared to the untreated condition, only for memory CD4TLs. This supports our hypothesis that the effects studied in this paper would be particularly relevant to the profiling of memory/effector T cells in peripheral organs in vivo.

### 3.7. Ex Vivo Association between MIF and Th17-like Cells in HIV Infection

**Figure 7:**
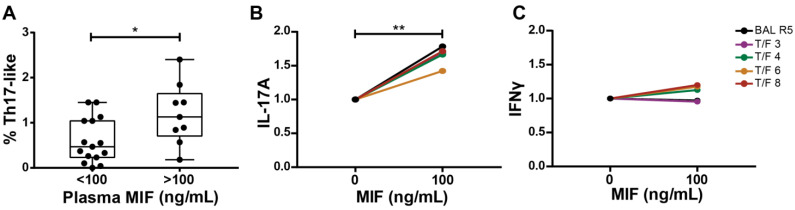
Association between MIF concentration in plasma and the proportion of IL-17A-expressing CD4TL in PBMCs, both derived from PWH. (**A**) Ex vivo IL-17A-expressing CD4TL percentage in PBMC from PWH segregated by low (<100 ng/mL) or high (>100 ng/mL) MIF plasma concentration. Data represent the median ± IQR25-75 of 15 donors. Data analysis was performed with a Mann–Whitney test. * *p* < 0.05. (**B**) Concentration of soluble IL-17A in MIF-stimulated co-cultures after five days of treatment with 100 ng/mL of MIF. MDM infection was performed with T/F HIV strains. Data show the average of means of at least three independent experiments with four different T/F HIV strains and the BAL R5 strain as a control, each one performed in triplicate, relative to the untreated control. Data analysis was performed with a Mann–Whitney test ** *p* < 0.01. (**C**) Concentration of soluble IFNγ in MIF-treated co-cultures after five days of treatment with 100 ng/mL of MIF with T/F HIV-strain-infected MDMs. Data show the average of means of at least three independent experiments with four different T/F HIV strains and the BAL R5 strain as a control, each one performed in triplicate, relative to the untreated control. Data analysis was performed with a Student’s *t*-test.

Finally, we set out to collect additional data from other models in order to provide further support to our findings. We decided to implement an ex vivo approach by using PBMCs from PWH. This approach provides with naturally infected cells and a context constituted not only by antigen-presenting cells and CD4TLs but also CD8TLs, B lymphocytes and NK cells. Proportions of IFNγ- and IL-17A-producing cells, HIV-infected cells and MIF plasma concentration were evaluated ex vivo in PBMC samples obtained from acutely infected PWH. We reasoned that an association between plasma MIF concentration and percentages of IL- 17A-expressing CD4TL would exist if MIF played a role in the activation and profiling of CD4TLs in vivo. In line with this, Figure 7A shows that higher percentages of bulk IL-17A-expressing CD4TLs were observed in samples with higher MIF plasma levels. In order to test if 100 ng/mL of MIF could replicate the findings observed with 25 ng/mL MIF, we decided to repeat the assay with T/F strains. As expected, 100 ng/mL MIF also induced a significantly higher secretion of IL-17A (Figure 7B) but not IFN-γ (Figure 7C) in HIV-infected MDM/CD4TL co-cultures, as shown in Figure 5 for 25 ng/mL MIF.

**Figure 8:**
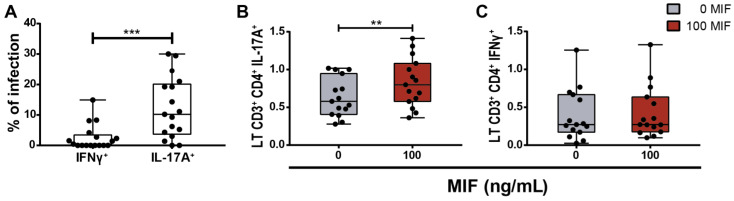
Evaluation of PBMCs from PWH response to MIF stimulus in an ex vivo model. (**A**) Ex vivo HIV-infection percentage in IL-17A- and IFNγ-expressing CD4TLs derived from PBMCs of PWH. (**B**) IFNγ intracellular expression on CD4TLs after 24 h of treatment with 100 ng/mL of MIF. (**C**) IL-17A intracellular expression on CD4TLs after 24 h of treatment with 100 ng/mL of MIF. Both experiments were performed on PBMCs and the results were standardized to the positive control (PBMCs treated with αCD3/αCD28-binding antibodies). Data represent the median ± IQR25-75 of 15 independent donors, each one with one replicate. Data analysis was performed with a Student’s *t*-test. ** *p* < 0.01, *** *p* < 0.001.

Then, we corroborated that the Th17-like subset contained a higher proportion of HIV-infected cells compared to the Th1 subset as previously described (Figure 8A). Next, we stimulated PBMCs from PWH with 100 ng/mL of MIF during 24 h. The time of stimulation was based on previous reports [42,43]. Then, percentages of IL-17A- and IFN-γ-producing cells were evaluated by flow cytometry. Non-stimulated and αCD3/αCD28-stimulated PBMCs served as negative and positive controls, respectively. Figure 8B,C show the results, standardized to the positive control. Figure 8B shows that the proportion of IL-17A-expressing CD4TLs was increased in the MIF-stimulated condition, compared to the untreated condition. On the other hand, IFNγ-expressing CD4TLs were not significantly different between conditions (Figure 8C). This reinforces the idea of a specific role of MIF in Th17-like profile differentiation.

## 4. Discussion

Signaling pathways triggered by MIF-CD74 interaction have an important role in the regulation of immune processes, inflammatory and autoimmune diseases, and cancer pathogenesis. However, research on its contribution to HIV immune dysfunction has been limited even in face of the evidence of CD74 and MIF overexpression during HIV-1 infection [14,15,16,18,20,21,44].

Recently, macrophage profile has gained great interest, leading to the identification of subpopulations with different functionalities [40]. It has also been described that HIV-1 infection induces a M1 polarization, an observation that has been broadly reported [45,46]. Additionally, what is more, our own group could characterize the short-term expression of pro-inflammatory cytokines by HIV-infected MDMs, in response to MIF stimulus after CD74 engagement [16].

The immune activation of MDMs can be studied mainly by evaluating membrane markers and cytokine expression as defining characteristics [40]. In this way, in this paper, we provide evidence that MIF stimulus on infected MDMs leads not only to the expression of pro-inflammatory cytokines, but also that this effect is accentuated over time (Figure 1A). In parallel, the anti-inflammatory cytokine IL-10 decreases, which enhances the inflammatory properties of the context. However, neither the expression of the evaluated membrane markers (CD80, CD86 and HLA-DR) (Figure 1B), nor the expression of other cytokines such as IL-4, IFNγ and IL-2 were altered in this model. Thus, this evidence points to an activation of HIV-infected MDMs in a M1-like manner after MIF stimulus.

It is widely known that immune context provided by cytokines constitutes a key factor during lymphocytic activation and profiling process, initiated by antigen recognition via TCR. In our co-culture experimental model, antigen recognition and co-stimulatory signals were provided by the infected MDMs. As MIF does not alter HLA-DR or co-stimulatory signal expression, the percentage of activated CD4TLs may not differ between conditions. Only cytokine concentration was found to be altered by MIF presence. Particularly, the cytokines found to be increased in MIF-treated HIV-infected MDM supernatants were IL-6 and IL-1β, in addition to a decrease in IL-10 concentration and no changes in that of IFNγ, leading us to speculate whether CD4TL activation could be skewed to a Th17-like phenotype [47,48].

When evaluating CD4TL activation profile, CD3^+^CD4^+^IL-17A^+^ events were augmented in MIF-treated cultures, but not CD3^+^CD4^+^IFNγ^+^ events (Figure 2A). Additionally, stimulated culture supernatants showed higher concentrations of soluble IL-17A and lower IFNγ compared to the control (Figure 2B). In agreement with this, RORγt-expressing CD4TLs were increased in MIF-treated cultures, but not Tbet-expressing CD4TLs (Figure 2C). Additionally, these Th17-like cells showed a higher susceptibility to HIV trans-infection driven by the infected MDMs present in the co-culture (Figure 3D). This is a characteristic of Th17 cells that we could also corroborate ex vivo in PBMCs from PWH (Figure 7A). As a whole, these results indicate that MIF stimulus might induce the expression of soluble factors, which in turn could promote a Th17-like phenotype in activated CD4TLs. In order to confirm that these cells are truly Th17 cells, this characterization should be complemented by the evaluation of other Th17-related cytokines, such as IL-17F, IL-21, IL-22 and IL-23, plus membrane markers such as CCR6. What is more, the expression of the HIV coreceptors (CCR5 and CXCR4) on the surface of these cells could also contribute to identify subpopulations within CD4 profiles. According to previous reports, high concentrations of IL-6 and IL-1β are important inducers of CD4TL activation into a Th17 profile [47]. These two cytokines are overexpressed by MDMs upon their stimulation with MIF (Figure 1A); interestingly, the increase in IL-6 is not as sharp when co-culturing the MDMs with CD4TLs (Figure 2D), which points to a consumption of this cytokine during the polarization process, as the internalization of IL-6 and its receptor is a necessary step for the activation of JAK/STAT3, and the later expression of RORγt [49,50,51]. On the other hand, Th1 profile co-stimulatory signals and CD4TL activation are not altered when incubating with MIF. This reinforces the idea that events following MIF triggering modify the CD4TL activation process at the profiling level and may impact only the Th17-like profile.

In this context, it was suggested as a possible hypothesis that blocking the MIF–CD74 interaction may lead to an abrogation of this CD4TL profiling process. Making use of two different tools, a chemical MIF antagonist and an MIF-neutralizing antibody, we intended to interfere with the receptor–ligand interaction. Both tools are specifically known to block the MIF interaction with CD74 [26]. In addition, it was interesting to evaluate the contribution of IL-6 and IL-1β to the process. Figure 4D shows how the percentage of Th17-like cells diminishes when MIF signaling was abrogated with both the MIF-neutralizing antibody and MIF chemical antagonist. This, together with our previously published results, supports the general hypothesis that MIF–CD74 triggering in HIV-infected MDMs has an impact on CD4TL profiling. However, using alternative and complementary tools to inhibit the MIF–CD74 interaction is needed to definitively confirm the role of this pathway in this model. This is the blocking of CD74 instead of MIF or the different molecules (CD44, CXCR2 and CXCR4) that have been described to form the heteromeric MIF receptor together with CD74.

On the other hand, IL-6 and IL-1β neutralization did not have the same effect. Although a decreased percentage of Th17-like cells was observed, it was not statistically different to the control. All in all, blocking MIF did the job, but impeding each cytokine activity individually did not. Other factors not accounted in this paper might be involved in the process, such as IL-23 and TGFβ, two possible soluble factors of importance in these results since they promote Th17 profiling [41,52,53].

Previously, the effect of IL-6 and IL-1β on CD4TL polarization has been reported [7]. Thus, our co-culture media could have the potential of inducing a Th17-like CD4TL profiling even in the absence of MDMs. Based on this last hypothesis, HIV-infected MDM culture supernatants were used to treat CD4TLs in parallel with an external stimulus (PHA or αCD3/αCD28-binding antibodies) in replacement of the activation signals provided by the infected MDMs. This experimental approach failed to replicate the previous results, as it can be seen in Figure 4A–C, highlighting the relevance of the unique conditions provided by the co-culture. First of all, the replacement of MDMs with either PHA or αCD3/αCD28 antibodies did not supply an antigen recognition signal via the TCR. This is the main signal that promotes CD4TL activation and, in this model, may have a crucial role in CD4TL polarization. On the other hand, there is a mechanism that cannot be replicated, which is cell-to-cell contact. Not only does it provide co-stimulatory signals that are not present in the new experimental model, but also the cell-to-cell contact that allows the viral transfer from MDMs to CD4TLs [54]. CD4TLs are susceptible to HIV infection; so, there is an active cell-to-cell trans-infection process in the co-culture, which is absent when not using MDMs as activation stimulus. Last but not least, viral replication in both cell types is a factor of relevance in the context of an immune response induction. Despite MDM supernatants containing HIV-1 viral particles, these are not as efficient in infecting CD4TLs as the trans-infection from infected MDMs.

Aiming to support these observations, we worked to obtain evidence by culturing PBMCs from PWH in the presence of MIF. In this ex vivo model, MIF plasma concentration showed a relation with IL-17A expressing CD4TLs. Higher MIF plasma levels (>100 ng/mL) were related to higher percentages of Th17-like CD4TLs in comparison with individuals with lower MIF plasma levels (<100 ng/mL) (Figure 7A). Moreover, the stimulation of these PBMCs with MIF (100 ng/mL) for 24 h led to an increase in IL-17A-expressing CD4TLs within the PBMCs. This concentration coincided with the cut off found in MIF plasma levels, which favor an augmented proportion of IL-17A^+^ CD4TLs. This supports the notion of an association between MIF and the promotion of a Th17-like profile, and it is in line with reports conducting similar assays using PBMCs from people with active Systemic Lupus Erythematosus or Rheumatoid Arthritis, where a bias to a Th17-like environment is observed after MIF stimulation [42,43].

In addition to its functional properties, the CD4TL profiles also differ on their permissiveness to HIV-1 infection. Particularly, Th17 cells are reported as one of the two most permissive populations. This could be corroborated in this cohort of PWH when comparing the infection percentages in Th1-like and Th17-like populations, finding higher values on the latter subset. Even more, this cellular subtype is supposed to play an important role on viral persistence [8]. The persistence of integrated HIV-1 genomes is stable in this particular population even under an effective HAART, which constitutes a significant contribution to viral reservoir maintenance [5,9].

The literature describes a Th17 population depletion in peripheral blood and tissues during HIV-1 infection [38,55,56]. At first, this seems contradictory with an MIF-dependent increase in IL-17A-expressing CD4TLs. However, we envisage that viral infection modulates the immune response promoting the activation of CD4TLs into a Th17-like profile and, subsequently, this cell becomes the main target for infection. This way, a sink–source system can be stablished. The equilibrium may be found at higher or lower Th17-like CD4TL percentage values, depending on MIF expression and other factors.

Nevertheless, it has to be taken into account that in, this experimental model, the antigen-presenting cell is an infected MDM, which is a limitation of this work. In a physiological context, the naïve CD4TLs are mainly activated by dendritic cells and not by MDMs, which, in turn, are present in primary lymphatic organs and present an adequate expression of co-stimulatory signals. T-cell profiling is, at present, considered a more flexible process. It is assumed that some cellular re-polarization exists in accordance with the predominant inflammatory micro-environment [57]. Particularly, the exchange proposed in this paper takes place between Treg and Th17 profiles. Both share TGFβ as an inductive stimulus but differ in which cytokine accompanies it: IL-10 for the Treg profile and IL-6 for the Th17 profile [57]. Interestingly, IL-6 also propitiates re-polarization processes in an unidirectional way (from Treg to Th17) [58]. Additionally, what is more important, this was one of the cytokines detected in infected-MDM supernatants in response to MIF stimulus. Thus, MIF (and, potentially, the MIF–CD74 interaction) could be a relevant factor in the CD4TL plasticity that T-cell profiles show in peripheral organs, where the effectors cells (infected MDMs) can be found. The fact that, in our model, both naïve (overall defined as CD45RA^+^CCR7 high) and memory CD4TLs showed increased proportions of Th17-like cells after MIF stimulus, but only in the memory CD4TL the difference was statistically significant (Figure 6), acquires particular relevance.

To sum up, MIF may have a role in modulating the adaptive immune response in the context of HIV-1 infection, raising highly permissive cell proportions, promoting viral spreading and contributing to the establishment and stability of viral reservoirs.

Methodologically, a strength of this work is the use of only primary cell cultures, resembling what happens in vivo better and allowing us to propose that MIF effects are observed even when accounting for donor variability. Additionally, replicating these results with transmitted-founder HIV-1 strains was one of our objectives, since these are clinically relevant viral strains. Last but not least, all MIF concentrations were physiologically relevant, resembling in vivo conditions.

## 5. Conclusions

In general, this work offered more information regarding the role of MDMs during HIV-1 infection, not only as cells capable of sustaining viral replication, but also as sources of soluble factors that promote viral dissemination. The evidence presented in this paper suggests that MIF interaction on HIV-infected MDMs could be implicated in viral persistence and inflammation, both key aspects of HIV immune pathogenesis.

The MIF–CD74 interaction is positively modulated by HIV-1 infection. In this paper, we postulated that its triggering may establish favorable conditions for viral replication, on the one hand, by generating an inflammatory micro-environment that increases viral replication, and on the other hand, by polarizing the CD4TL response towards a Th17-like phenotype that is more permissive to HIV-1 infection.

These data lay the foundations for further studies in order to delve deeper into the study of the MIF–CD74 interaction as a future therapeutic target to reduce inflammation and viral replication and to determine its possible role in HIV viral reservoirs.

## Data Availability

The data presented in this study is available upon request to the corresponding author.

## Supplementary Materials

The following supporting information can be downloaded at: https://www.mdpi.com/article/10.3390/v14102218/s1. Figure S1: Representative gating of (A) CD4 T cells and (B) p24 staining of CD4 T cells co-cultured with infected MDMs treated and untreated with MIF. (C,D) Expression of cytokines IL-17A and IFN-γ and transcription factors RORγt and Tbet in CD4^+^ T lymphocytes co-cultured with HIV-infected MDMs for 5 days in the presence of MIF (25 ng/mL) or not. Dot plots from one representative experiment are shown.
