# Supplementary material for: Macrophage Migration Inhibitory Factor (MIF) Promotes Increased Proportions of the Highly Permissive Th17-like Cell Profile during HIV Infection
Source: Viruses. 2022 Oct 9;14(10):2218. doi: 10.3390/v14102218 (PMC9611675; doi:10.3390/v14102218)
Supplement: Supplementary file 1 [file viruses-14-02218-s001.zip › viruses-1910719-supplementary.pdf]

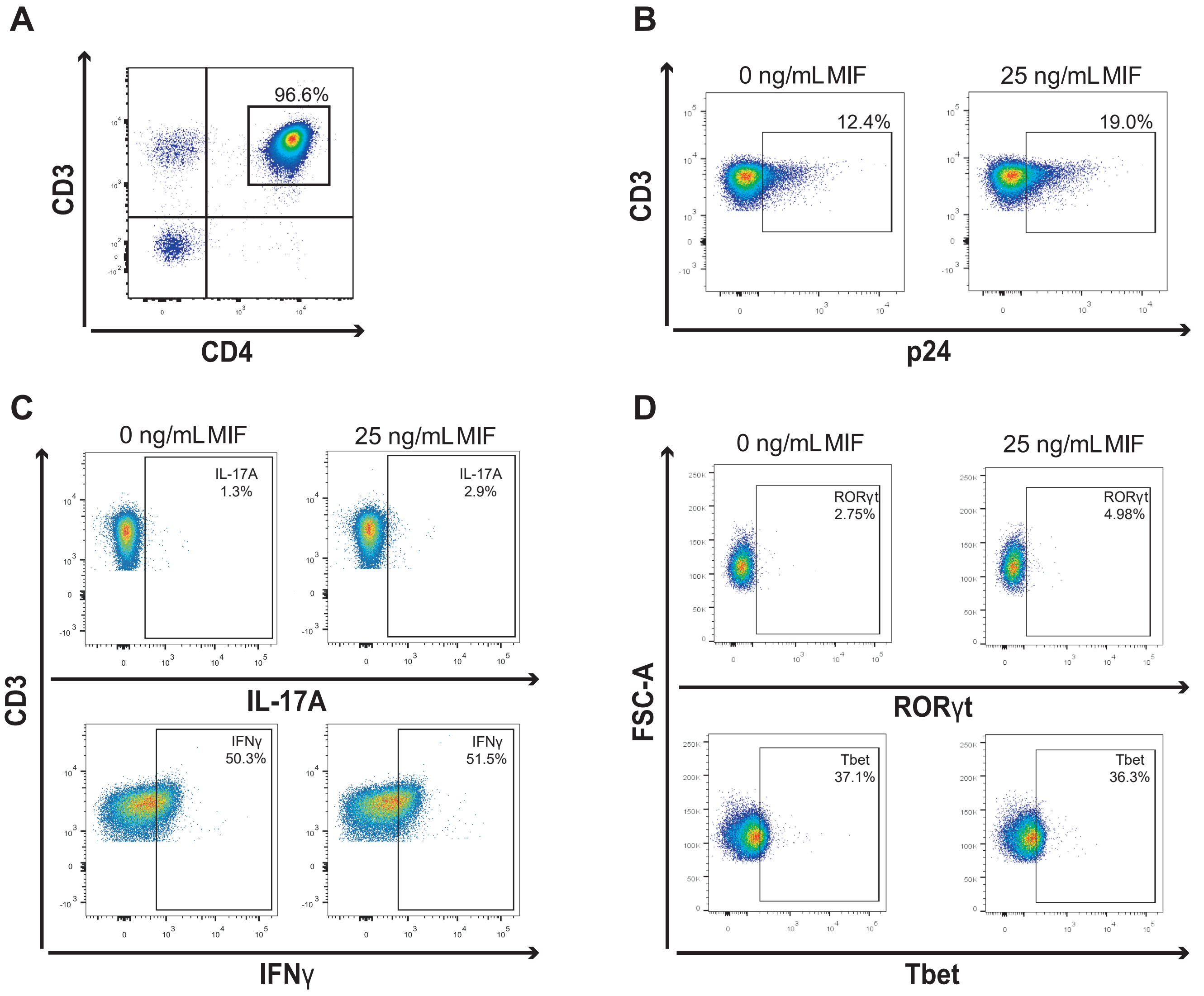

Figure S1: Representative gating of (A) CD4 T cells and (B) p24 staining of CD4 T cells co-cultured with infected MDMs treated and untreated with MIF. (C,D) Expression of cytokines IL-17A and IFN-γ and transcription factors RORγt and Tbet in CD4+ T lymphocytes co-cultured with HIV-infected MDMs for 5 days in the presence of MIF (25 ng/mL) or not. Dot plots from one repre-sentative experiment are shown.
